# Towards precision medicine in vascular anomalies: Could protein kinase C inhibitors be repurposed for GNAQ/11‐related phakomatoses?

**DOI:** 10.1111/srt.13736

**Published:** 2024-06-04

**Authors:** Yaron Gu, James P. Pham, Deshan F. Sebaratnam

**Affiliations:** ^1^ Faculty of Medicine University of New South Wales Kensington New South Wales Australia; ^2^ Department of Dermatology Liverpool Hospital Liverpool New South Wales Australia; ^3^ Laboratory of Translational Cutaneous Medicine Ingham Institute of Applied Medical Research Liverpool New South Wales Australia

Dear Editor

Many of the targeted therapies employed in vascular anomalies have been propagated from oncology experience because of overlap with pathogenic variants in solid tumours. A prime example is the phosphatidylinositol 3‐kinase (PI3K) inhibitor alpelisib, developed for PIK3CA‐mutated breast cancer, repurposed for PIK3CA‐related overgrowth syndrome following proof‐of‐concept murine models and a single‐arm clinical trial.^1^

GNAQ/GNA11 encode for Gαq/Gα11—alpha subunits of the heterotrimeric G proteins, Gq and G11—which activate a diverse array of signal transduction pathways such as PI3K, non‐canonical Hippo signalling through Rho/Rac as well as downstream signalling through protein kinase C (PKC).^2^ PKC isoforms regulate cellular proliferation, differentiation, survival and angiogenesis via downstream stimulation of the mitogen‐activated protein kinase (MAPK) pathway (Figure 1). GNAQ/GNA11 variants have been identified in 90% of primary uveal melanoma and PKC inhibitors such as darovasertib are demonstrating promise in this cancer which was previously associated with poor survival outcomes.^3^

**FIGURE 1:**
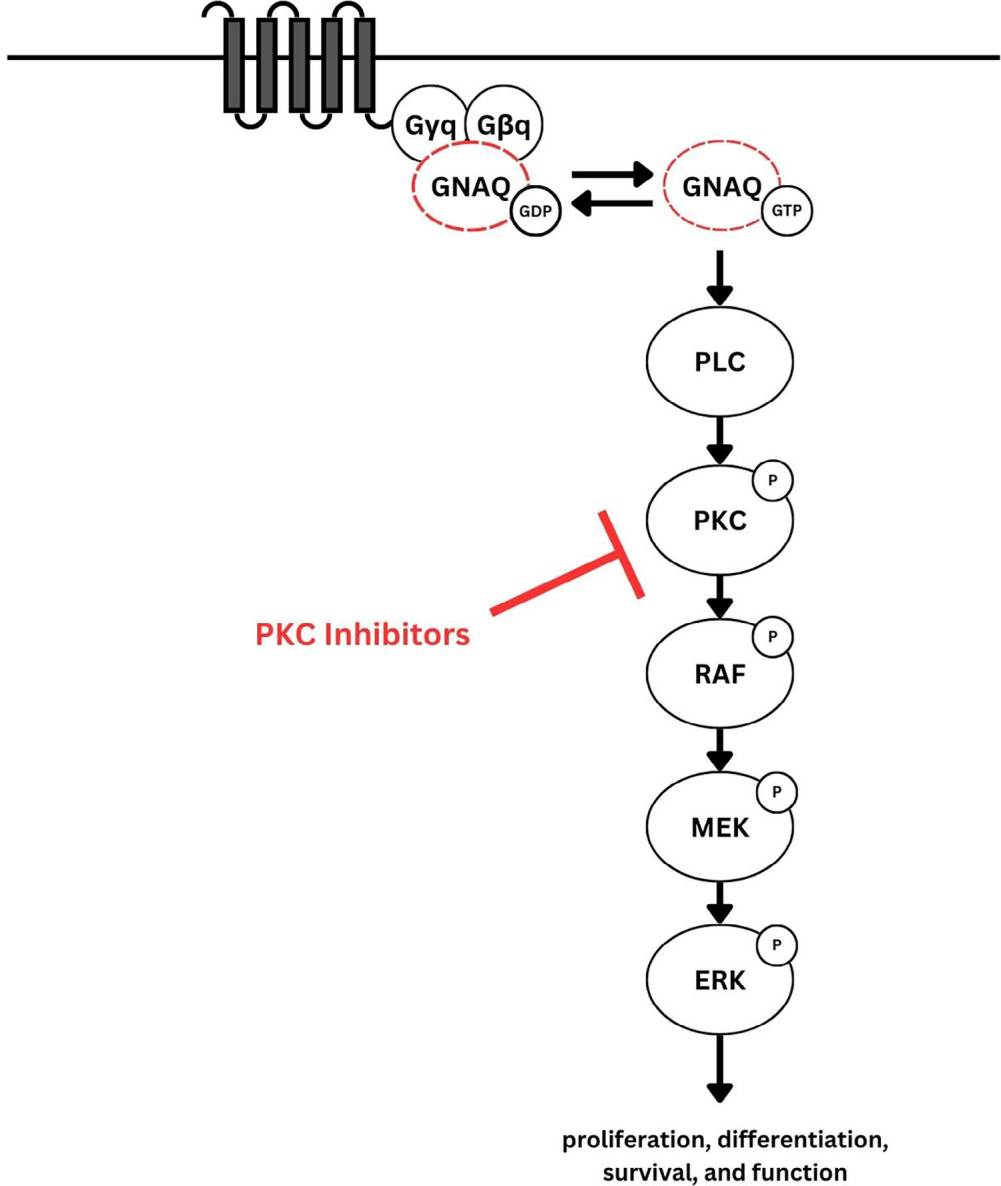
Genetic mutation in GNAQ leads to hyperactivation of downstream molecular pathways and dysregulation of endothelial cell function in Sturge‐Weber Syndrome.^2^ ERK, extracellular signal‐regulated kinases; MEK, mitogen‐activated protein kinase kinase; PKC, protein kinase C; PLC, phospholipase C; RAF, Rapidly accelerated fibrosarcoma.

The somatic variants GNAQ c.548G > A and GNA11 c.547C > T have also been identified in several vascular anomalies such as capillary malformation, congenital haemangioma and phakomatosis pigmentovascularis. In a recent ex‐vivo study, phosphorylated PKC‐α was increased in hypertrophic capillary malformations compared to adjacent skin, further suggesting this pathway may be implicated in the pathogenesis of this disorder.^4^ We speculate that in future, PKC inhibitors might play a potential role in the management of vascular anomalies.

For example, somatic activating variants of GNAQ have been found in approximately 80% of lesional skin, brain and scleral tissue in patients with Sturge‐Weber syndrome (SWS).^2^ Rather than multiple interventions for each affected organ system (pulsed dye laser, anti‐glaucoma eye drops, anti‐epileptic medications, etc.), a single drug that targets the aberrant pathways across organ systems offers parsimony.

Drug repurposing offers several advantages including previously established safety data and the mitigation of costs, time and attrition associated with new drug discovery. Despite early evidence of effect in GNAQ/GNA11 mutated cancers,^3^ further preclinical ‘proof of concept’ validation is essential before the repurposing of PKC inhibitors in SWS or similar GNAQ/GNA11‐dependent vascular anomalies. To date, the bulk of our knowledge concerning GNAQ/11 in SWS has been gleaned from genome sequencing and histological analysis of patient tissue samples. At present, in vivo models do not effectively mimic SWS biology in humans and no complex in vitro SWS model exists yet.^2^ However, recent advances in disease models such as microphysiological systems have shown promise in studying other complex vascular malformations in vitro.^5^ Validating the utility of PKC inhibition in vascular anomalies may include studying PKC expression (including its various isoforms) in GNAQ‐mutant models; downstream molecular effects following PKC knockout; and cellular responses to PKC blockade, prior to in‐human clinical trials.

It is important that clinicians are aware of recent advances in oncogene translational research, as these may provide potential precision medicine opportunities for our patients in the future.

## CONFLICT OF INTEREST STATEMENT

Deshan F. Sebaratnam has received consulting fees from Galderma, AbbVie, Amgen, Pfizer, Novartis, Janssen, Leo Pharma, Ego Pharmacy, Viatris, Sun Pharma and material support from Candela Medical and Heine Optotechnik. Yaron Gu has received a scholarship from Ego Pharmaceuticals. James P. Pham has no conflicts to report.

## Data Availability

Data sharing is not applicable to this article as no datasets were generated or analysed during the current study.

## References

[1] Venot Q , Blanc T , Rabia SH , et al. Targeted therapy in patients with PIK3CA‐related overgrowth syndrome. Nature. 2018;558(7711):540‐546. doi:10.1038/s41586-018-0217-9 29899452 PMC7610773

[2] Van Trigt WK , Kelly KM , Hughes CCW . GNAQ mutations drive port wine birthmark‐associated Sturge‐Weber syndrome: a review of pathobiology, therapies, and current models. Front Hum Neurosci. 2022;16:1006027. doi:10.3389/fnhum.2022.1006027 36405075 PMC9670321

[3] Piperno‐Neumann S , Carlino MS , Boni V , et al. A phase I trial of LXS196, a protein kinase C (PKC) inhibitor, for metastatic uveal melanoma. Br J Cancer. 2023;128(6):1040‐1051. doi:10.1038/s41416-022-02133-6 36624219 PMC10006169

[4] Yin R , Gao L , Tan W , et al. Activation of PKCα and PI3K kinases in hypertrophic and nodular port wine stain lesions. Am J Dermatopathol. 2017;39(10):747‐752. doi:10.1097/dad.0000000000000785 28030367 PMC5478501

[5] Ewald ML , Chen YH , Lee AP , Hughes CCW . The vascular niche in next generation microphysiological systems. Lab Chip. 2021;21(17):3244‐3262. doi:10.1039/d1lc00530h 34396383 PMC8635227

